# 

**Published:** 1858-11

**Authors:** 


PUBLISHED MONTHLY, AT $3 PER ANNUM IN ADVANCE.

### ATLANTA MEDICAL AND SURGICAL JOURNAL.

EDITED BY

JOSEPH P. LOGAN, M. D.,

PROFESSOR OF PHYSIOLOGY AND GENERAL PATHOLOGY IN THE ATLANTA MEDICAL COLLEGE.

AND

W. F. WESTMORELAND, M. D.,

PROFESSOR OF THE PRINCIPLES AND PRACTICE OF SURGERY IN THE ATLANTA MEDICAL COLLEGE.

Pax et scientia, sed veritas sine timore.

VOL. IV. NOVEMBER, 1858. NO. III.

ATLANTA, GEORGIA:
PRINTED BY J. I. MILLER & CO.
(SUCCESSORS TO G. P. EDDY & CO.)
1858.

EACH NUMBER CONTAINS SIXTY-FOUR PAGES.

